# Repeated Long-Term Sub-concussion Impacts Induce Motor Dysfunction in Rats: A Potential Rodent Model

**DOI:** 10.3389/fneur.2020.00491

**Published:** 2020-05-29

**Authors:** Andrew P. Lavender, Samuel Rawlings, Andrew Warnock, Terry McGonigle, Bailey Hiles-Murison, Michael Nesbit, Virginie Lam, Mark J. Hackett, Melinda Fitzgerald, Ryusuke Takechi

**Affiliations:** ^1^School of Health and Life Sciences, Federation University, Ballarat, VIC, Australia; ^2^School of Physiotherapy and Exercise Science, Faculty of Health Sciences, Curtin University, Perth, WA, Australia; ^3^Curtin Health Innovation Research Institute, Curtin University, Perth, WA, Australia; ^4^Perron Institute for Neurological and Translational Science, Ralph and Patricia Sarich Neuroscience Research Institute, Nedlands, WA, Australia; ^5^School of Pharmacy and Biomedical Sciences, Faculty of Health Sciences, Curtin University, Perth, WA, Australia; ^6^School of Public Health, Faculty of Health Sciences, Curtin University, Perth, WA, Australia; ^7^School of Molecular and Life Science, Faculty of Science and Engineering, Curtin University, Perth, WA, Australia; ^8^Experimental and Regenerative Neurosciences, School of Biological Sciences, The University of Western Australia, Perth, WA, Australia

**Keywords:** beamwalk, mild traumatic brain injury, neuromotor function, rotarod, sub-concussion

## Abstract

Whilst detrimental effects of repeated sub-concussive impacts on neurophysiological and behavioral function are increasingly reported, the underlying mechanisms are largely unknown. Here, we report that repeated sub-concussion with a light weight drop (25 g) in wild-type PVG rats for 2 weeks does not induce detectable neuromotor dysfunction assessed by beamwalk and rotarod tests. However, after 12 weeks of repeated sub-concussion, the rats exhibited moderate neuromotor dysfunction. This is the first study to demonstrate development of neuromotor dysfunction following multiple long-term sub-concussive impacts in rats. The outcomes may offer significant opportunity for future studies to understand the mechanisms of sub-concussion-induced neuropsychological changes.

## Introduction

Non-concussion or “sub-concussive” head impact is defined as “a cranial impact that does not result in known or diagnosed concussion on clinical grounds, which may be the result of a slosh phenomenon” (1). However, an increasing number of clinical studies report that when the sub-concussion is repeated over a long-term, it results in substantial neurological and neuropsychological alterations (1). For instance, studies in soccer players demonstrated that the frequency of ball headings correlated with structural changes of the brain (2, 3). However, very few studies to date investigated the effects of repeated sub-concussion in animal models (4), and its underlying mechanisms remain largely unknown. One of those studies by Shultz et al. showed that a single sub-concussion impact by mild lateral fluid percussion injury (0.50–0.99 atm) resulted in no neurobehavioral changes, whilst elevated neuroinflammation was evident (5). Although the mechanical force of sub-concussion and duration of sub-concussive impacts in rats are not directly translatable to humans, this study presented no behavioral or clinical changes in the rats, falling into the aforementioned definition of sub-concussion. Furthermore, a study by Xu et al. used a weight-drop model to compare the effects of sub-concussion induced by different weights of 20, 40, and 60 g (6). The study found that the increasing weight corresponded with increasingly severe traumatic axonal injury in optic nerves, corpus callosum and cerebellum, without showing significant motor deficits. However, these studies only used a single sub-concussive impact, and do not represent frequently repeated sub-concussive impacts over a prolonged period of time.

Thus, in the present study, we employed lighter sub-concussive impacts with a 25 g weight-drop in wild-type PVG rats, which was 10-fold lighter than our previous study where more severe form of concussion was demonstrated (7). The weight drop was repeated multiple times (10 impacts/d, 3 d/week) for an extended period (2 or 12 weeks). The weight of 25 g was chosen because the previous study by Xu et al. demonstrated that 20–60 g weight drop provides sub-concussion-like impacts in rats (6). In addition, our pilot studies found that unlike the previously described procedure with 250 g weight (7), the rats receiving 25 g weight drop did not fall through the hole in the stage of the weight-drop device to create body rotation movement, thus the body and head remained on a holding paper towel. The latter is ideal for representing minor head knocks with minimum or no spinal movement/whiplash injury, which are the lighter end of sports-associated concussive impacts. Due to its substantial differences in biology and physiology, the frequency and duration of rat's sub-concussion procedure are not directly translatable to human. Nonetheless, a previous study by Agoston reports that based on longevity differences, one rat day is approximately equivalent to 27 human days, whilst other measures such as metabolic rate, protein turnover and heart rate present significantly different timelines between rats and humans (8). Moreover, the paper indicates that such timeline can substantially vary depending on the life stage (e.g., adulthood vs. adolescence). For simplicity, we used the longevity based equation to estimate an approximate human equivalent duration and frequency of our sub-concussion procedure as summarized in Table 1.

**Table 1:** Summary of sub-concussion protocol.

	**Procedure**	**Age**	**Number of impacts per**	**Number of procedure**	**Total procedure**	**Total impacts**
	**duration**		**session per day**	**days per week**	**days**	**received**
Rat procedure	2 weeks	6–8 weeks	10 impacts	3 days	6 days	60 impacts
(Human equivalent^*^)	(0.9 years)	(2.8–3.8 years)	(N/A)	(40.5 days)	(81 days)	(N/A)
Rat procedure	12 weeks	6–18 weeks	10 impacts	3 days	36 days	360 impacts
(Human equivalent^*^)	(5.7 years)	(2.8–8.5 years)	(N/A)	(40.5 days)	(972 days)	(N/A)

* *Based on 1 rat day = 27 human days (8)*.

Thereafter, we examined the effects of the long-term repeated sub-concussive impacts on neuromotor performance and tested whether the duration of the sub-concussion period impacted on any significant differences.

## Materials and Methods

### Animals

A total of 40 female PVG rats at 5 to 6 weeks old were purchased from Animal Resources Centre (WA, Australia) and were randomly allocated to sub-concussion group (SC) or Sham group. Female rats were selected to be consistent with our previous studies of concussion (7, 9). Young rats were chosen to be consistent with clinical findings, where soccer ball headings in childhood appear to induce neuronal impairment (3). The rats were maintained on standard maintenance chow purchased from Specialty Feeds (WA, Australia, Meat Free Rat and Mouse Diet). All animal procedures in this study were approved by the Curtin University Animal Ethics Committee (ARE2019-13/19).

### Sub-concussion and Sham Procedure

**Figure 1:**
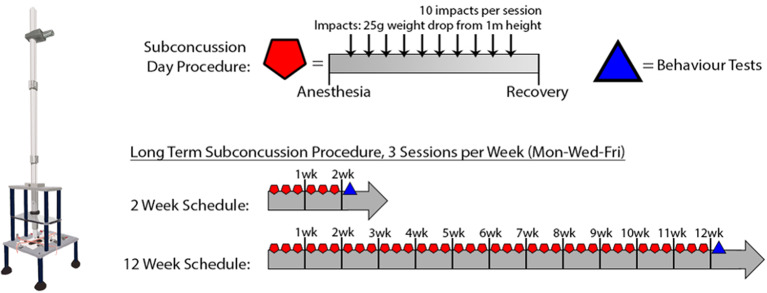
Summary of Sub-concussion Protocol.

Sub-concussive impacts were administered to the SC group rats with a custom-built weight-drop device (Northeast Biomedical, MA, USA) as described previously with minor modification (weight changed from 250 to 25 g) (7). Briefly, a 25 g weight was dropped from 1 m height to the impact site on lambda (2–3 mm anterior to the front of ears) under 3% isoflurane anesthesia. The weight drop was administered 10 times consecutively, with ~10–30 s between each impact and after each impact, the impact site was re-aligned to lambda. The series of 10 SC impacts was repeated 3 days per week (i.e., Monday/Wednesday/Friday) for a period of 2 or 12 weeks. Sham group rats received 3% isoflurane without the weight drop for the same duration as SC procedure. After the SC or Sham procedure, the rats were left on a heating pad until recovered from anesthesia before returning to their home cage. There was no prolonged loss of consciousness beyond the normal duration of anesthesia recovery. For summary, please refer to Figure 1 and Table 1.

### Neuromotor Function Tests

Following 2 or 12 weeks of repeated sub-concussion impacts, neuromotor function was assessed within 48 h of sub-concussion procedure completion by utilizing established beamwalk and rotarod tests as previously described with some minor modifications (9–11). Briefly, for beamwalk, a beam of 1, 2, or 3 cm width and 30 cm length, raised 30 cm above the floor was used. The rats were placed at one end of the beam facing toward the other end, where their home cage was located. Each rat was given 3 trials on each 3, 2, and 1 cm width beam. A mean number of foot slips and latency to reach the home cage (by two front paws reaching the home cage wall) were recorded.

The rats were acclimatized on a Rotarod apparatus (Orchid Scientific, India), rotating at 4 rpm for 60 s. At least a day of acclimatization was done with three trials per day until the rats stay on the rod without falling for 60 s. On the test day, the rats were placed on the rotating rod, accelerating from 4 to 40 rpm within 300 s. The rotation speed and latency time until the rats fell from the rod was recorded and the mean of three trials was used.

### Statistical Analyses

All data are expressed as mean with standard error of the mean. Data normality was assessed with D'Agostino & Pearson test on Prism 8 (GraphPad Software, CA, US). In order to compare the neuromotor function between Sham and SC rats at each time point, unpaired *t*-tests were used for normally distributed data whilst non-parametric Mann-Whitney tests were used for the data that were not normally distributed. Statistical significance was considered to be *p* < 0.05.

## Results

**Figure 2:**
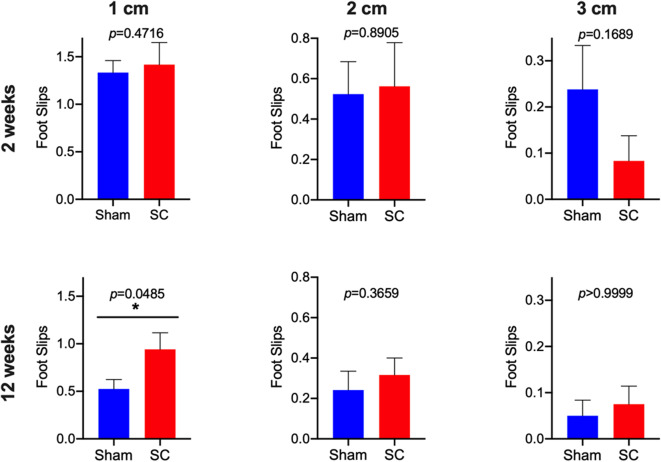
Beamwalk Test. Neuromotor function was tested by using beamwalk test with 1, 2, and 3 cm beams in Sham and Sub-concussion (SC) group rats after 2 or 12 weeks of repeated sub-concussions. The number of foot slips are presented as a mean ± SEM of 3 trials. Statistically significant differences between Sham and SC groups at each time point was tested with unpaired *t*-test or non-parametric Mann-Whitney test (*p < 0.05).

**Figure 3:**
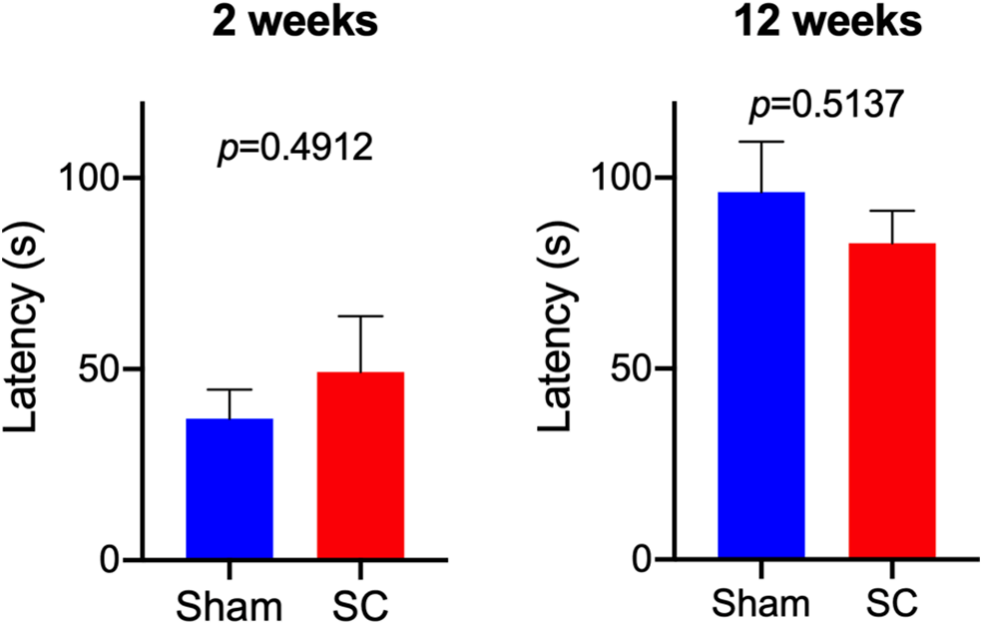
Rotarod Test. Rotarod test was used to compare the neuromotor performance between Sham and Sub-concussion (SC) group rats following 2 or 12 weeks of repeated sub-concussions. The mean latency that the rats were able to stay on the accelerating rod was expressed as a mean ± SEM of 3 trials. Statistical significance was assessed with unpaired *t*-test or non-parametric Mann-Whitney test.

After receiving repeated sub-concussive head impacts for 2 weeks, the number of beamwalk foot slips whilst crossing the 3, 2, or 1 cm wide beam was comparable with the rats that received Sham procedure for 2 weeks (Figure 2). The latency that SC rats stayed on the accelerating rotarod was also not significantly different from the Sham group after 2 weeks of repeated sub-concussion impacts (Figure 3).

When the rats were given sub-concussion impacts repeatedly for 12 weeks, the SC rats showed significantly greater number of foot slips on the 1 cm width beam, compared with the Sham group rats (Figure 2). Whereas, a comparable number of beamwalk foot slips were observed between SC and Sham rats on the 3 and 2 cm wide beams. The rotarod test latency between the SC and Sham rats was not significantly different (Figure 3).

## Discussion

The present study is the first to test the effects of sub-concussive impacts that are repeated multiple times over an extended period, on neuromotor function in wild-type PVG rats. After 2 weeks of repeated sub-concussive head impacts, the rats showed no significant deterioration in neuromotor function, assessed by beamwalk and rotarod tests, although SC rats on 3 cm beam showed a non-significant trend of decreased foot slips. However, when the repeated sub-concussion was given for 12 weeks, the rats demonstrated significant neuromotor dysfunction when compared with the Sham group rats. This neuromotor deficit was only detectable with the narrow 1 cm beamwalk, whilst the results from wider 2 cm and 3 cm beamwalks and rotarod were comparable to the age-matched Sham rats. In beamwalk tests, the narrower beams are considered to be more sensitive in detecting motor deficits, in comparison to the wider beams (12). Moreover, beamwalk tests are reported to offer higher sensitivity over rotarod tests (13). These data collectively indicate that only after 12 weeks of repeated sub-concussive impacts, rats begin to show moderate but significant motor dysfunction, and further suggests that extended sub-concussion periods >12 weeks may induce further deterioration of neuromotor performance. The latter is consistent with clinical studies where neurobehavioral performance may only be affected by repeated sub-concussion over a prolonged term. McAllister et al. reported that collegiate contact sports players in football and ice hockey performed significantly poorer in neuropsychological and neurocognitive tests than control group athletes participating in non-contact sports (14). It is worthwhile noting that the athletes who participated in this study had no documented sports concussion during the period of study. On the other hand, Miller et al. showed that in collegiate football players, no differences in computerized neuropsychological test performance were observed between pre-season, mid-season, and post-season assessments (15), indicating that it is likely that more than one season period is required for the neurocognitive decline to become evident.

It was also noted that in the current study, that after 12 weeks of sham procedures, rats showed generally improved neuromotor function in beamwalk and rotarod tests, compared with 2 weeks Sham rats. This may result from the age of the rats when the neuromotor assessments were done, irrespective of the duration of Sham or SC procedure. However, these data were beyond the scope of the current study and may be considered further in future.

In conclusion, the current study was the first to demonstrate that in rats, frequent repeated sub-concussive impacts over an extended period, greater than 2 weeks and detectable at 12 weeks, induce modest neuromotor dysfunction. The findings provide evidence that 25 g weight drop in PVG rats provide a novel sub-concussion experimental model that may represent clinical long-term cumulative sub-concussion impacts. The advantage of this novel model is that it potentially represents clinical findings where neurostructural and neuromotor dysfunction are demonstrated following repeated sub-concussive events (e.g., soccer player's headings). A great majority of previous studies with animal models of traumatic brain injuries adopt more severe types of injuries, which are not appropriate to represent “sub-concussion.” Thus, the model may be utilized to investigate relevant mechanisms and the establishment of therapeutic strategies for sub-concussion-associated neuromotor deficits in sports athletes.

## Data Availability Statement

All datasets generated for this study are included in the article/supplementary material.

## Ethics Statement

The animal study was reviewed and approved by Curtin Animal Ethics Committee.

## Author Contributions

This study was designed and managed by AL, SR, VL, MH, MF, and RT. The animal maintenance, procedure, and sample/data collection were done by AL, SR, AW, TM, BH-M, MN, VL, MH, MF, and RT. The data interpretation and manuscript preparation were done by AL, SR, VL, MH, MF, and RT.

## Conflict of Interest

The authors declare that the research was conducted in the absence of any commercial or financial relationships that could be construed as a potential conflict of interest.

## References

[1] Mainwaring L Ferdinand Pennock KM Mylabathula S Alavie BZ. Subconcussive head impacts in sport: a systematic review of the evidence. Int J Psychophysiol. (2018) 132(Pt. A):39–54. 10.1016/j.ijpsycho.2018.01.007 29402530

[2] Rodrigues AC Lasmar RP Caramelli P. Effects of soccer heading on brain structure and function. Front Neurol. (2016) 7:38. 10.3389/fneur.2016.00038 27047444 PMC4800441

[3] Koerte IK Lin AP Willems A Muehlmann M Hufschmidt J Coleman MJ. A review of neuroimaging findings in repetitive brain trauma. Brain Pathol. (2015) 25:318–49. 10.1111/bpa.12249 25904047 PMC5699448

[4] Shultz SR McDonald SJ Vonder Haar C Meconi A Vink R van Donkelaar P. The potential for animal models to provide insight into mild traumatic brain injury: translational challenges and strategies. Neurosci Biobehav Rev. (2017) 76(Pt. B):396–414. 10.1016/j.neubiorev.2016.09.014 27659125

[5] Shultz SR MacFabe DF Foley KA Taylor R Cain DP. Sub-concussive brain injury in the Long-Evans rat induces acute neuroinflammation in the absence of behavioral impairments. Behav Brain Res. (2012) 229:145–52. 10.1016/j.bbr.2011.12.015 22245525

[6] Xu L Nguyen JV Lehar M Menon A Rha E Arena J. Repetitive mild traumatic brain injury with impact acceleration in the mouse: multifocal axonopathy, neuroinflammation, and neurodegeneration in the visual system. Exp Neurol. (2016) 275(Pt. 3):436–49. 10.1016/j.expneurol.2014.11.004 25450468

[7] Yates NJ Lydiard S Fehily B Weir G Chin A Bartlett CA. Repeated mild traumatic brain injury in female rats increases lipid peroxidation in neurons. Exp Brain Res. (2017) 235:2133–49. 10.1007/s00221-017-4958-8 28417146

[8] Agoston DV. How to translate time? The temporal aspect of human and rodent biology. Front Neurol. (2017) 8:92. 10.3389/fneur.2017.00092 28367138 PMC5355425

[9] Mao Y Black AMB Milbourn HR Krakonja S Nesbit M Bartlett CA. The effects of a combination of ion channel inhibitors in female rats following repeated mild traumatic brain injury. Int J Mol Sci. (2018) 19:3408. 10.3390/ijms19113408 30384417 PMC6274967

[10] McGill JK Gallagher L Carswell HV Irving EA Dominiczak AF Macrae IM. Impaired functional recovery after stroke in the stroke-prone spontaneously hypertensive rat. Stroke. (2005) 36:135–41. 10.1161/01.STR.0000149629.32525.b7 15569870

[11] Abada YS Nguyen HP Schreiber R Ellenbroek B. Assessment of motor function, sensory motor gating and recognition memory in a novel BACHD transgenic rat model for huntington disease. PLoS ONE. (2013) 8:e68584. 10.1371/journal.pone.0068584 23874679 PMC3708912

[12] Sawers A Ting LH. Beam walking can detect differences in walking balance proficiency across a range of sensorimotor abilities. Gait Posture. (2015) 41:619–23. 10.1016/j.gaitpost.2015.01.007 25648493

[13] Luong TN Carlisle HJ Southwell A Patterson PH. Assessment of motor balance and coordination in mice using the balance beam. J Vis Exp. (2011) 10:2376. 10.3791/2376 21445033 PMC3197288

[14] McAllister TW Flashman LA Maerlender A Greenwald RM Beckwith JG Tosteson TD. Cognitive effects of one season of head impacts in a cohort of collegiate contact sport athletes. Neurology. (2012) 78:1777–84. 10.1212/WNL.0b013e3182582fe7 22592370 PMC3359587

[15] Miller JR Adamson GJ Pink MM Sweet JC. Comparison of preseason, midseason, and postseason neurocognitive scores in uninjured collegiate football players. Am J Sports Med. (2007) 35:1284–8. 10.1177/0363546507300261 17405886

